# Schizophrenia as Failure of Left Hemispheric Dominance for the Phonological Component of Language

**DOI:** 10.1371/journal.pone.0004507

**Published:** 2009-02-18

**Authors:** Alessandro Angrilli, Chiara Spironelli, Thomas Elbert, Timothy J. Crow, Gianfranco Marano, Luciano Stegagno

**Affiliations:** 1 Department of General Psychology, University of Padova, Padova, Italy; 2 CNR Institute of Neuroscience, Padova, Italy; 3 Center for Psychiatry, Reichenau and Department of Psychology, University of Konstanz, Konstanz, Germany; 4 SANE POWIC, Department of Psychiatry, Warneford Hospital, Oxford, United Kingdom; 5 Center for Eating Disorders, ULSS 18, Rovigo, Italy; University of Granada, Spain

## Abstract

**Background:**

T. J. Crow suggested that the genetic variance associated with the evolution in *Homo sapiens* of hemispheric dominance for language carries with it the hazard of the symptoms of schizophrenia. Individuals lacking the typical left hemisphere advantage for language, in particular for phonological components, would be at increased risk of the typical symptoms such as auditory hallucinations and delusions.

**Methodology/Principal Findings:**

Twelve schizophrenic patients treated with low levels of neuroleptics and twelve matched healthy controls participated in an event-related potential experiment. Subjects matched word-pairs in three tasks: rhyming/phonological, semantic judgment and word recognition. Slow evoked potentials were recorded from 26 scalp electrodes, and a laterality index was computed for anterior and posterior regions during the inter stimulus interval. During phonological processing individuals with schizophrenia failed to achieve the left hemispheric dominance consistently observed in healthy controls. The effect involved anterior (fronto-temporal) brain regions and was specific for the Phonological task; group differences were small or absent when subjects processed the same stimulus material in a Semantic task or during Word Recognition, i.e. during tasks that typically activate more widespread areas in both hemispheres.

**Conclusions/Significance:**

We show for the first time how the deficit of lateralization in the schizophrenic brain is specific for the phonological component of language. This loss of hemispheric dominance would explain typical symptoms, e.g. when an individual's own thoughts are perceived as an external intruding voice. The change can be interpreted as a consequence of “hemispheric indecision”, a failure to segregate phonological engrams in one hemisphere.

## Introduction

In 1877 Paul Broca [1] wrote that – “Man is, of all the animals, the one whose brain in the normal state is most asymmetrical. He is also the one who possesses most acquired faculties. Amongst these faculties… the faculty of language holds pride of place. It is this that distinguishes us most clearly from the animals.” Broca's conjecture is supported by anatomical evidence that the cerebral “torque” from right frontal to left occipital [2] in the human brain and its correlate of asymmetry to the left in the minicolumn structure of the planum temporale [3] is absent in the brain of the chimpanzee. Directional asymmetry may thus be the characteristic that defines the brain of *Homo sapiens* and the anatomical correlate of language [4].

Schizophrenia is a psychiatric disorder with an onset (earlier in males) in the reproductive phase of life present in all populations with an approximately uniform lifetime prevalence of 0.8% [5]. Consistent with the concept that this is an anomaly of hemispheric lateralization [6] are the presence of structural [7]–[12] as well as functional [13] deviations of hemispheric asymmetry present in the majority of individuals in the normal population. Reviewing the evidence, Crow [14], [15] suggested that the genetic variance associated with the evolutionary development, in *Homo sapiens*, of dominance for a component of language in one hemisphere carries with it the hazard of failure manifest as the symptoms of schizophrenia. Thus, in any population a small but approximately uniform fraction of individuals will fail to develop the characteristic left hemisphere advantage for the critical component of language as a consequence of an unfavorable endowment of species-specific variation. The consequent ectopic emergence of this component in the non-dominant hemisphere would explain core symptoms such as auditory hallucinations in which the individual's own verbal engrams (in one language centre) are perceived as external voices/thoughts intruding through activation of inter-hemispheric connections on the other centre. According to this concept, Crow [14], [15] concluded that the core deficit is disruption of the left-hemispheric shift for language, especially those components relating to phonological articulation, a distinct linguistic process located in left dorso-lateral pre-frontal cortex. On the basis of electrophysiological and metabolic techniques a few studies have yielded evidence for altered lateralization in schizophrenia of tones, syllables [16], [17] or words [18]–[22]. However, these studies have not demonstrated that the loss or anomaly of asymmetry in schizophrenia is specific to phonological processing by comparison with other components of language. The aim of the present experiment was to examine language lateralization in a group of patients with schizophrenia, by contrasting three relevant functions: phonological, semantic and lexical. We expected the Phonological task to be the most left lateralized in healthy controls. The paradigm manipulates the task rather than the linguistic stimuli, and is based on recordings of language-related slow brain potentials. Unlike early potentials (P200, P300, N400), slow potentials are quite large waves generated by wide superficial cortical layers and their negativity is a relatively unambiguous index of cortical activation [23]–[25]. Furthermore, our CNV paradigm adapted for language has been previously validated in different languages [23], in aged subjects [26], and it has been successfully applied to language lateralization in dyslexic [27] and aphasic [28], [29] patients.

## Methods

### Participants

Psychiatric assessment of schizophrenics has been performed by using both clinical standard instruments (DSM IV TR) and quantitative evaluation by means of the Andreasen's Scale [30], [31]. The diagnosis classified three patients as disorganized, five patients with residual, two with paranoid, two with undifferentiated schizophrenia. The sample was characterized by relatively high levels of Flattened affect (mean±SD: 18.75±11.2) and Anhedonia (mean±SD: 20.75±5.3) as negative symptoms and relatively high rating of Delusions (mean±SD: 14.83±6.2) on the scale of positive symptoms (see Table 1). The twelve selected patients (six women) had mean age 37.9 (SD: ±11.3; range 24–60), mean education 12.3 years (SD: ±2.9), and were compared with 12 healthy matched controls (six women) mean age 35.8, (SD: ±13.5; range 25–61; t_22_ = 0.41, p = 0.69), and mean education 14.9 years (SD: ±4.1; t_22_ = 1.83, p = 0.08).

**Table 1:** Patients' demographic data along with Diagnosis, Medication, Andreasen's scales for negative and positive symptom assessment.

					SANS					SAPS			
Patient	Age	Sex	Type	Medication	F. AFF.	AL.	AP.	AN.	ATT. I.	HALL.	DEL.	BIZ./D. B.	P. F. T. D.
F.G.	51	M	Disorgan.	Perphenazine enantate 8 mg/d	6	0	6	10	6	10	4	11	22
				Chlorpromazine 50 mg/d									
D.D.	41	F	Undiffer.	Perphenazine enantate 6 mg/d	5	7	12	20	13	0	0	0	31
L.S.	39	M	Residual	Haloperidol 3 mg/d	29	13	10	23	10	8	11	3	4
C.A.	49	M	Disorgan.	Perphenazine enantate 4 mg/d	12	0	6	20	7	0	15	17	11
B.N.	27	F	Residual	Haloperidol 2 mg/d	26	20	18	25	13	12	20	12	13
B.I.	60	F	Undiffer.	Haloperidol decanoate 2 mg/d	7	0	7	23	6	9	13	11	27
Z.T.	23	F	Residual	Clotiapine 20 mg/d	30	20	15	24	12	0	13	0	0
P.G.	31	M	Residual	Clotiapine 10 mg/d	26	20	15	25	9	12	25	0	0
C.M.	24	M	Paranoid	Fluphenazine decanoate 2.5 mg/d	6	5	8	21	9	14	20	13	29
P.L.	36	F	Disorgan.	Perphenazine enantate 2 mg/d	26	10	9	23	9	0	13	5	0
P.R.	33	F	Residual	Levomeprazine maleate 25 mg/d	16	11	17	10	6	0	13	17	15
V.T.	41	M	Paranoid	Haloperidol 1 mg/d	36	15	20	25	9	0	17	0	13
Mean					18.75	10.08	11.92	20.75	9.08	5.42	14.83	7.42	11.47

**TYPE:** Disorgan. = Disorganized; Undiffer. = Undifferentiated. **SANS:** F.AFF. = Flattened Affect; AL. = Alogia; AP. = Apathy; AN. = Anhedonia; ATT. I. = Attentional Impairment. **SAPS:** HALL. = Hallucinations; DEL. = Delusions; BIZ./D. B. = Bizzarre or Disorganized Behavior; P.F.T.D. = Positive Formal Thought Disorder.

Patients were treated with about one third of the standard neuroleptic dosage (Table 1): with an average daily medication of 70 mg chlorpromazine-equivalents, range: 20–130 mg. All subjects were right handed with a percentage of right hand dominance ranging 90 to 100% as measured with the Edinburgh Inventory [32]. All participants were monolingual and native speakers of Italian. The experimental procedure was approved by the Ethics Committee of the Faculty of Psychology, University of Padova. Subjects participated in the electrophysiological session after giving their written informed consent according to the Declaration of Helsinki. Psychiatrists treating the patients explained them the experimental procedure and ensured their mental competence in understanding and giving written informed consent to participate.

### Apparatus and physiological recordings

Electroencephalographic traces were collected continuously in Direct Current (DC) mode, by following the main requirements for high quality DC recordings [33], with bandwidth ranging from DC to 30 Hz (6 dB/octave), sampling rate of 100 Hz, and amplitude resolution of 0.1 µV/bin. Impedance was kept below 5 KΩ. Electrical signals were measured by means of 26 tin electrodes, using the DCMES 32-channel system (MES, Münich), 19 mounted on an elastic cap (ElectroCap) according to the International 10–20 system [34]; the other seven electrodes were applied below each eye (Io1, Io2), on the two external canthi (F9, F10), on the Nasion (Nz, i.e., at the intersection of the frontal and nasal bones) and on mastoids (M1, M2). The vertex (Cz) was used as on-line recording reference for the EEG channels, then data were converted off-line and referred to the mean across all channels, i.e., converted to average reference.

### Stimuli, tasks and procedure

Words and pictures of objects served as visual stimuli. Line drawings of objects representing concrete and frequent words were selected from the collection of Snodgrass and Vanderwart [35]. Verbal stimuli consisted of bi- or trisyllabic Italian content words selected from a frequency dictionary of 5000 written Italian words [36], and words with emotional content were not included (words are listed in Appendix S1). Particular care was given to the selection of word pairs: specific semantic categories (such as living, non-living, tools, animals, foods, and so on) were not contrasted, but rather equivalent levels. Stimuli were presented in pairs on a 17″ computer monitor one at a time, with an interstimulus interval (ISI) of 2 seconds (sec): the first (W1) was always a word and remained on the screen for 1 sec, the second stimulus (target) was a word (Phonological and Semantic tasks) or a picture (Word Recognition task) which was visually presented for up to 5 sec until the subject responded by pressing a keyboard button. Pairs were associated with three tasks in separate blocks: during Phonological and Semantic tasks, the same words being presented as W1 in a different randomized order [23], [26]. The subject was instructed to decide whether the second word (or the picture in the Word Recognition task) did or did not match the first word according to a rhyme judgment (Phonological task; e.g. cut-hut) semantic categorization (Semantic task; e.g. tooth-tusk), lexical decision (Word Recognition; e.g. the word “window” followed by a picture representing a window). Participants had to respond to the target stimulus, i.e., after the presentation of each pair of stimuli, by pressing a keyboard button with the 2^nd^ or 3^rd^ finger of the left hand [further details are described in ref. 23]. Each task included 80 trials/stimulus-pairs and lasted about 10 min. In all tasks, 50% matched and 50% mismatched trials varied in pseudorandom order. The order of the tasks was randomly varied across subjects.

The paradigm adopted, based on language-related slow brain potentials, has been previously validated in healthy subjects speaking Italian and German [23], in aged healthy subjects [26], and it has been successfully used to evaluate language lateralization in aphasics [28], [29]. The Phonological task was of special interest as it elicits activity typically localized in the left hemisphere at the fronto-temporal level [37], [38], and this lateralization was predicted to be abnormally distributed in schizophrenia [15]. In previous experiments based on such paradigms, the relative cortical negativity, termed Contingent Negative Variation (CNV), which develops during the interval between the two contingent stimuli, is the sum of all extracellular dendritic post-synaptic potentials produced at the level of the apical cortical layers [24], [25], and it is functionally inhibited by underlying cortical lesions in humans [28], [29].

### Data Analysis

Error Rates (ERs) and Response Times (RTs) of each subject served as behavioral measures, and mean performance was compared between groups and tasks.

Data were re-referenced off-line to average values, and epoched into 13-sec intervals including 1 sec before and 12 sec after W1 onset. A 100-msec baseline preceding W1 was subtracted from the whole trial epoch. Single trials were corrected for vertical and horizontal eye movements and blinking artifacts, using BESA software (Brain Electrical Source Analysis, 5.1 version) to compute an ocular correction coefficient [39]. Each trial was then visually inspected for potential residual artifacts. Contaminated trials were excluded from further analyses. The MSEC correction method ascertained high fidelity of the data analyzed [39], especially for the activity in the frontal regions that is typically distorted or underestimated in traditional eye correction procedures. Therefore, also orbitofrontal electrodes (Fp1, Fp2, F9, F10, Nz, Io1, Io2) can be considered to measure activity from artifact free active cortical sites.

After eye movement correction, all trials with correct responses were averaged for each task and for each subject. In agreement with our past study [26], after the visual inspection of waveforms, three functional intervals corresponding to specific phases of word elaboration have been chosen for data analysis: the 500 msec preceding the first word offset (W1 condition), the first sec time window after the offset of the first word (corresponding to the initial Contingent Negative Variation; *i*CNV condition), and the last sec after the offset of the first word (corresponding to the terminal Contingent Negative Variation; *t*CNV condition) were analyzed. For these three intervals, a laterality score was computed as the difference of the mean activity of right (electrodes: Fp2, F8, Ve2, F10) minus left (electrodes: Fp1, F7 Ve1, F9) anterior quadrants, and a similar lateralization was computed for posterior sites, right (electrodes: T8, P4, P8, O2) minus left posterior quadrants (electrodes: T7, P3, P7, O1).

The laterality score is positive when activity (i.e., negativity) is left lateralized and negative when it is right lateralized. The statistical analysis of variance (ANOVA) included, for every temporal window, the following factors: Group (Schizophrenic Patients vs. Controls), Brain Region (Anterior vs. Posterior), Task (Word Recognition vs. Phonological vs. Semantic).

With regard to behavioral measures (mean ERs and RTs), the ANOVA included the between-subjects Group factor (two levels: Schizophrenic Patients vs. Controls) and the within-subjects Task factor (three levels: Word-Picture matching vs. Phonological vs. Semantic).

Post hoc comparisons were computed, whenever the null hypothesis was rejected, in order to test in which conditions the means of each group differed significantly. We used the Newman Keuls test (p<0.05), a post hoc test which is already corrected for multiple comparisons, and the Greenhouse-Geisser index which allows correction for ANOVA statistics when sphericity assumptions are not completely fulfilled (this correction is used to adjust p value when df are >1).

## Results

### Behavioral data

**Figure 1:**
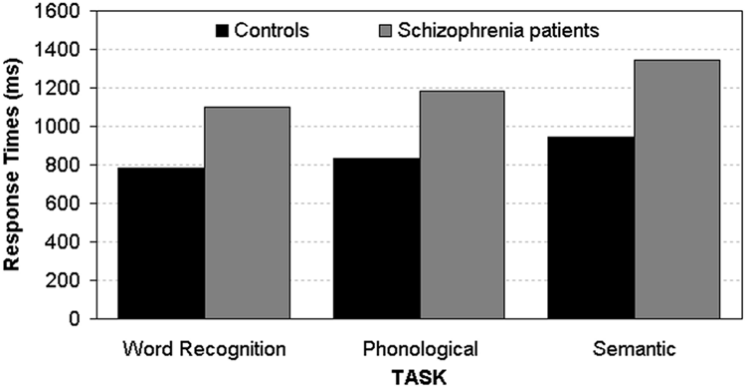
Mean Response Times (RTs) showed by controls and schizophrenic patients in the three linguistic tasks.

The Group main effect (F_1,22_ = 15.04, p<0.001) showed that patients were slower to respond than controls (mean: 1209±262 vs. 854±228 ms, respectively; Figure 1). In addition, both groups were faster in Word-Picture matching than in Phonological than in the Semantic task (mean: 942±260 and 1008±328 vs. 1145±289 ms, respectively; Task main effect: F_2,44_ = 21.18, p<0.001, ε = 0.93).

Analysis of ERs showed only a Group main effect (F_1,22_ = 5.74, p<0.05), which revealed that patients made more errors than controls (mean: 9.02±9.76% vs. 3.19±2.23%, respectively).

### Electrophysiological data

#### First interval – W1 condition (0.5 sec preceding the first word offset)

**Figure 2:**
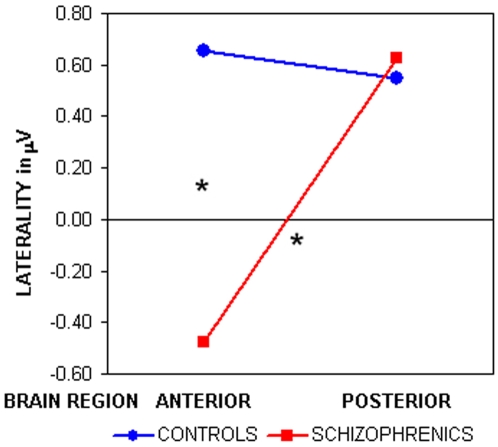
Lateralization score, measured as the difference of scalp activity from electrodes of the right hemisphere minus the activity of the left homologous electrodes, are shown for anterior and posterior quadrants. Positive values indicate left lateralization; activity was measured in the 0.5- to 1-sec interval after word onset (W1 condition).

ANOVA computed on late phases of word reading (W1 condition) revealed a significant Task main effect (F_2,44_ = 4.30, p<0.01, GG ε = 0.99), showing that, independently from groups, the Phonological task was more left lateralized than the Semantic task (−0.67±1.56 vs. 0.02±1.63 µV respectively, p = 0.014). The two-way Group by AP asymmetry interaction (F_1,22_ = 4.83, p<0.05) evidenced for controls a significant left lateralization both on anterior and posterior regions (Figure 2), whereas schizophrenic patients resulted significantly right lateralized over anterior areas in comparison with the left lateralization of posterior sites (p = 0.026). Thus, patients differed from controls only on their anterior locations (p = 0.038), while showed a regular left activation, compared to controls, over posterior clusters (Figure 2).

#### Second interval – iCNV condition (1 sec after W1 offset)

**Figure 3:**
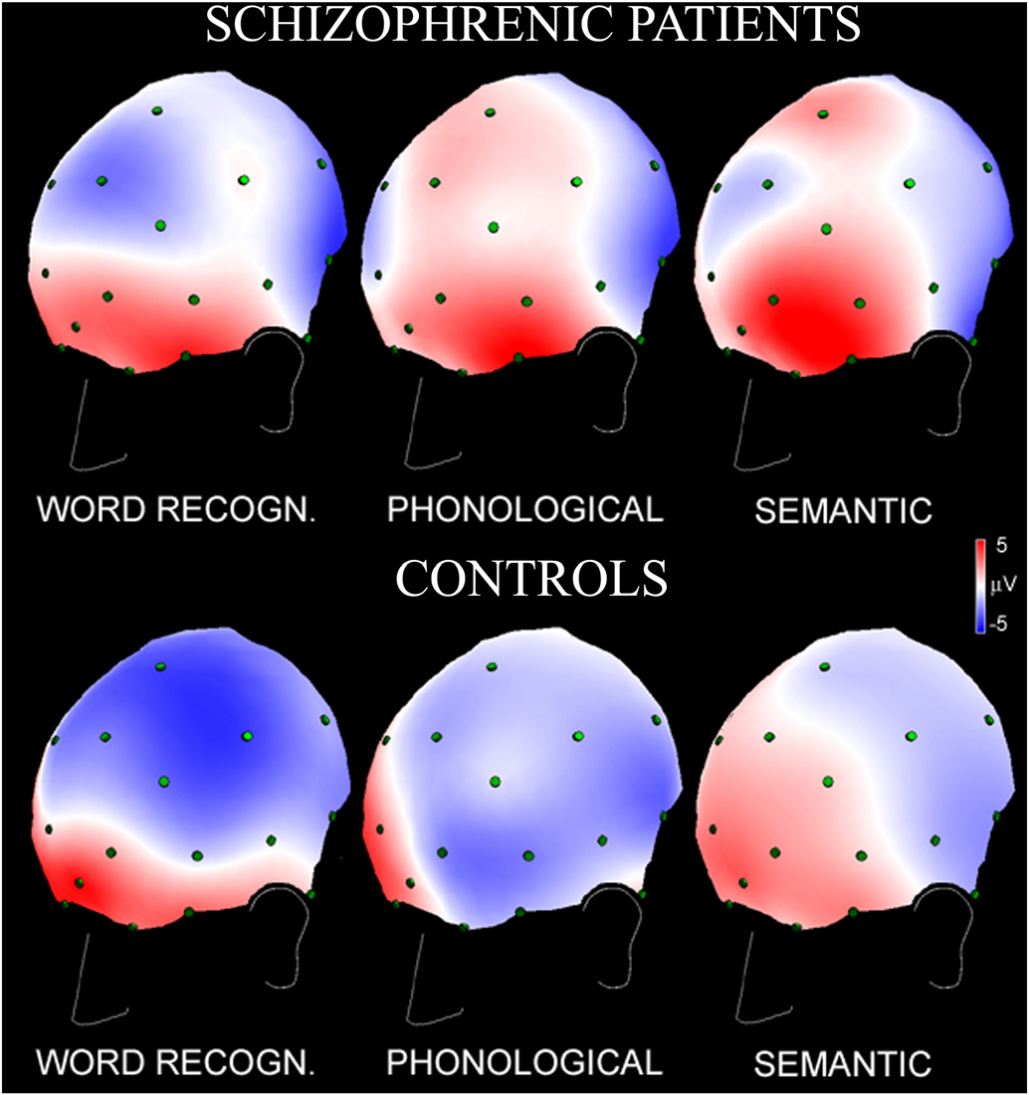
Spline maps of scalp electrical activity (3/4 left frontal view evidencing areas involved in phonological elaboration) measured in the first second of the inter stimulus interval (*i*CNV), for the three linguistic tasks. Patients in the upper panel, controls in the lower panel. Negativity, in blue, indicates activation of cortical layers in this language-related CNV paradigm, positivity, in red, indicates relative inhibition.

Spline maps of scalp electrical activity measured in the initial CNV, show an overall left frontal activation-negativity in controls in all tasks and a relative inhibition-positivity in schizophrenics (Figure 3). The effect was most pronounced for the Phonological task in which controls exhibited the largest left frontal activation and schizophrenics instead had inverted hemispherical pattern.

ANOVA of laterality scores confirmed the qualitative analysis. The significant Group main effect (F_1,22_ = 6.39, p<0.01) revealed that controls were overall more left lateralized than schizophrenic patients (−0.72±1.62 vs. 0.31±1.71 µV, respectively). As during W1 condition, the two-way interaction Group by Brain Region resulted significant (F_1,21_ = 13.3, p<0.001). While there was virtually no difference between groups over posterior areas, the lateralization for individuals with schizophrenia differed markedly from those of controls at fronto-temporal sites. The Newman-Keuls post-hoc test pointed to an overall greater relative activation of left frontal cortex in controls and relative inhibition of this area in schizophrenia (p = 0.0003). Furthermore, patients with schizophrenia exhibited inverted laterality at anterior as compared to their own posterior sites (p = 0.011).

**Figure 4:**
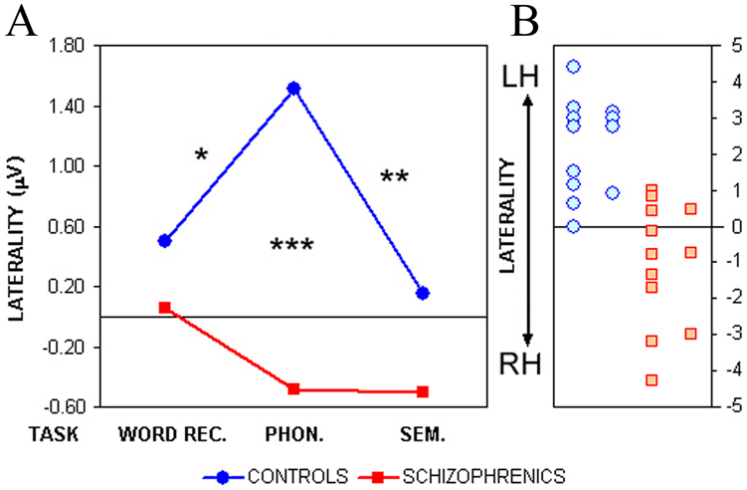
Lateralization scores (right minus left hemisphere electrical activity) during *i*CNV interval (*A*) in controls and schizophrenics for the three tasks. (*B*) Distribution of individual laterality scores from anterior sites in the Phonological task.

The significant two-way interaction Group×Task (F_2,42_ = 4.0, p<0.05, GG ε = 0.99) showed a specific effect of the task on the laterality of the two groups (Figure 4*A*).

Controls displayed significantly greater left lateralization during the Phonological in comparison with both Word Recognition and the Semantic tasks (Newman-Keuls post-hoc tests, p = 0.020 and p = 0.007 respectively). The latter two tasks activated cortical networks relatively widespread in the two hemispheres.

**Figure 5:**
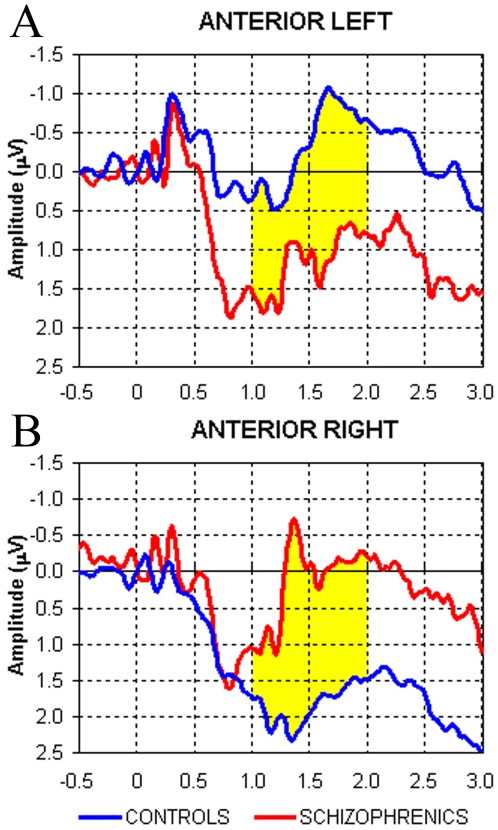
Time course of slow brain potentials from (*A*) left, and (*B*) right, anterior quadrants in the Phonological task. Waveforms represent the grand-averaging of the two samples of subjects (controls in blue, schizophrenics in red), the interval corresponding to the *i*CNV used for statistical analysis is in yellow. Positivity indicates relative cortical inhibition and negativity (upward) relative cortical activation. Patients were relatively less activated than controls on left anterior locations and relatively more activated on right locations.

The time course of electrical activity in the Phonological task gave evidence of greater negativity/activation over left anterior sites in controls and reversed laterality in patients (Figure 5*A* and 5*B*). On individual comparisons, patients showed bilateral or even inverted lateralization relative to controls (p = 0.0003; Figure 4*B*).

#### Third interval – tCNV condition (last sec after W1 offset)

The last interstimulus interval analyzed, termed tCNV, was expected to involve word rehearsal in the working memory and preparation of the motor response/decision. This interval was marked by a significant Group main effect (F_1,22_ = 11.21, p<0.01), which showed left lateralization in controls (−0.83±1.95 µV), and right lateralization in schizophrenics (0.55±2.13 µV). Furthermore, the two-way Group×AP gradient interaction (F_1,22_ = 8.17, p<0.01) revealed that groups differed again on anterior locations (p = 0.003), with controls left lateralized and patients right lateralized, whereas they showed overlapped activity over posterior regions. Moreover, schizophrenics had a significant greater lateralization at anterior than posterior areas (p = 0.025).

## Discussion

The main deficit in schizophrenia was localized at anterior left sites and, among the linguistic processes here investigated, was expressed as a significant inversion of asymmetry in the phonological component of words. Interestingly, the significant absence of left lateralization highlighted for the Phonological task appeared during a specific phase of word processing, the iCNV, an interval which is not well reflected by slower metabolic methods, such as fMRI and PET. The initial CNV, corresponding to the 1- to 2-sec epoch following W1 offset, has been clearly explained as the electrophysiological index of cognitive operations closely related to stimulus encoding [40], [41] in verbal working memory [24]. Furthermore, although the statistical method does not allow precise localization of electrical activity, the gross subdivision between anterior and posterior brain regions is valid [24]: electrodes placed on anterior quadrants of each hemisphere roughly collect activity from anterior temporal lobe, lateral prefrontal cortex and orbitofrontal-cingulate regions. These regions, especially the left parahippocampal gyrus [10] and the prefrontal cortex, and their connections, are the brain structures highlighted as the focus of pathology in schizophrenia [42], [43]. A cellular basis in the frontal lobes for the present findings is suggested by the observation, in post-mortem brain, of asymmetries of pyramidal cell density, size and shape, in area 9 of dorsolateral pre-frontal cortex [12]. In controls, cell density and size were greater on the left, and the cell bodies were more spherical vs. triangular in outline: in patients with schizophrenia the density was greater on the right and the asymmetries of size and shape were lost. In line with this observation, a study based on diffusion tensor imaging has demonstrated a loss of asymmetry in schizophrenia for the uncinate fasciculus [11]. This bundle of axons connects frontal cortex with the temporal pole and a relative reduction in the left hemisphere might be expected to lead to a loss of interconnectivity between these regions relevant to the deficits we have observed over the left hemisphere and located frontally in the phonological component of language. Functional deficits of left anterior cortical regions comparable to those in the present investigation were reported in a recent fMRI study that contrasted verbal with spatial working memory and showed in patients a loss of leftward asymmetry in the inferior frontal cortex [21].

In the present study for the first time we show that the deficit in schizophrenia was specific to the phonological formulation of words relative to their recognition and semantic interpretation, both of which were similarly lateralized in the two groups. In particular, the Semantic task represents a precise control condition as the same words activated different anatomical regions in the Phonological task. The result is in line with a body of literature [43]–[45] documenting anomalies of language related functions in the left hemisphere in schizophrenia but in the present research, by contrast with previous findings, the anomalies have been demonstrated to be selective to the phonological processing of words relative to other components of language.

In this investigation we controlled several variables that can confound electrophysiological observations on clinical populations. First, we selected patients with minimal neuroleptic medication. Second, a paradigm was chosen to facilitate examination of slow brain potentials. Pre-attentional and attentional deficits in schizophrenia, which are not central to the disease process, grossly modify the amplitudes of shorter latency evoked potentials, such as N100, N200 and P300 [13], [18]. Our paradigm avoided the confound of these early ERP components. Behavioral results further support this interpretation as patients were overall slower, but showed the same pattern across tasks exhibited by controls; furthermore, in agreement with the low medication, their error rates were quite small (less than 10%). The slow evoked potential that we assessed following the offset of the first word was not only unaffected by possible variations in early evoked components but also indexed activation of the large interconnected cortical networks of the left hemisphere that are involved in semantic and phonological components of verbal working memory [23], [26].

Previous experiments with presentation of simple sounds or syllables have yielded evidence within sensory areas of the temporal cortex consistent with the failure of lateralization hypothesis [16], [17]. The present investigation, by the use of slow evoked potentials [24] together with more complex linguistic stimuli that activate extended cortical areas [23], [25], [27]–[29] has more accurately documented the extent of the cortical networks recruited by word elaboration in the left hemisphere and their disruption in schizophrenia. Our findings are in agreement with the general view of schizophrenia as a deficit of integrative cortical function and loss of neural interconnectivity rather than as a disorder due to a specific functional-anatomical or neurochemical impairment [42], [46], [47].

Current research on the neural basis of language indicates that the left frontal cortex, especially Broca's area, plays a fundamental role in the organization of language as a whole (including the domains of semantics, syntax and phonology) as well as plans for action [48], [49], through its hierarchical influence over right frontal and left posterior linguistic areas. This concept is supported by extended review of fMRI and PET literature [50], [51] but also by experiments on patients with Broca's aphasia in whom language areas are reorganized as close as possible to undamaged left frontal regions [28], [29]. This suggests that language and thinking are integrated with other processes within a unique hierarchical system with its center located in the left frontal region. A similar, less severe, lack of integration between hemispheres is observed in developmental dyslexia [52], in which reading difficulty depends on loss of left frontal linguistic dominance [53], [54] and leads to a deficit in word segmentation and timing [55]. Further evidence for the parallel is provided by the observation that reading is delayed in pre-psychotic children [56]. In line with the most specific pathophysiological theory of schizophrenia [14], [15], [57], our patients lacked the typical left anterior dominance exhibited by healthy controls, and were characterized, on PANS, by relatively high level of delusions and thought disorders, with only a small subgroup of five patients showing hallucinations (table 1). In Crow's hypothesis with its foundation in evolutionary theory, positive symptoms (hallucinations, delusions and thought disorder) are related to loss of language asymmetry (see next paragraph for details). In this first experiment patients were selected mainly for minimal pharmacological treatment to limit the confound of medication: it was predicted that all patients with schizophrenia in general would be characterized by a loss of linguistic phonological asymmetry. In future experiments, the issue of the symptom correlates of lateralization will be addressed by selecting patients with predominant positive symptoms. Considering alternative explanations, it is also possible that loss of language lateralization is an epiphenomenon of psychosis: in this case language represents a good marker of loss of integration among a number of more basic processes and areas in schizophrenics' brain. However, such an interpretation would have to take account of the specificity of the deficits to the phonological task amongst other linguistic processes.

### Conclusions

Our results point to the conclusion that the primary change in schizophrenia is failure of lateralization to the left hemisphere of the phonological component of language. Taken together with the Broca-Annett axiom [4] that the cerebral torque is the characteristic that defines the human brain this suggests that schizophrenia is “the price that *Homo sapiens* pays for language” [14], [15]. According to this concept, the capacity for language evolved relatively abruptly 150 to 200,000 years ago as a result of a saltational genetic change that established the cerebral torque or modified an asymmetry that had entered earlier in the hominid lineage, with the consequence that the human brain is four-chambered (right and left frontal, and left and right parieto-temporo-occipital) [14], [15], [58]. One of the most interesting genetic candidates for the abrupt onset of language and for human hemispheric asymmetry is the *Protocadherin 11X/Y* gene, the relevance of which is suggested by its role in axonal guidance during brain development and its evolutionary history across species [59].

Hemispheric specialization enabled the segregation of “thought” and plans, in right dorso-lateral prefrontal cortex, from the generation of speech (associated with the phonological process) in Broca's area and surrounding dorso-lateral association cortex in the left hemisphere. Schizophrenic symptoms are what happens when at a late stage of neural development in adult life, as we have shown, the process of lateralization of the phonological component to the left hemisphere fails. We speculate that the segregation of function on which the coherence of language depends breaks down. Thoughts that are normally autonomous and strictly under the individuals control are experienced as influenced by the environment (thought insertion and withdrawal) and unconfined to the individual (thought broadcast [45], [58]). Neural engrams (of thoughts and plans for action – engram is defined as the neurophysiological correlate of a memory trace in the brain) that are self-generated are experienced as perceived speech. These engrams are identified by the affected individual as “thoughts spoken aloud”, and voices commenting on his actions because they activate phonological engrams in Wernicke's area and surrounding association cortex. Each of these “first rank” symptoms we see as potential consequences of incomplete lateralization of the phonological component of language to the dominant hemisphere.

On this view, what are described as the symptoms of the disease schizophrenia represent a disintegration of the components of language, or “language at the end of its tether”. They thus reflect a component of genetic variation that relates to the transition (speciation event) that gave rise to *Homo sapiens* as a species. The paradox that a component of the variation is disadvantageous (schizophrenia is associated with a substantial fecundity deficit) but is not selected out of the population was identified by Huxley and colleagues [60]. These evolutionary theorists overlooked the possibility that the genetic variation is *sapiens*-specific or that the balancing advantage that they sought was a clue to the origin of the species. We concur with Paul Broca that asymmetry and the capacity for language are features that distinguish ourselves from other primate and mammalian species. We conclude that variations in hemispheric dominance for the phonological component of language cast some light on the genetic origins of the species as well as the nature and origin of psychotic symptoms.

## Supporting Information

Appendix S1 (0.08 MB DOC)
