# Supplementary material for: Schizophrenia as Failure of Left Hemispheric Dominance for the Phonological Component of Language
Source: PLoS One. 2009 Feb 18;4(2):e4507. doi: 10.1371/journal.pone.0004507 (PMC2637431; doi:10.1371/journal.pone.0004507)
Supplement: Appendix S1 — (0.08 MB DOC) [file pone.0004507.s001.doc]

**APPENDIX S1.** Complete list of written Italian words used in the experimental paradigm. Note that the first sample of words (W1) was used in both Phonological and Semantic tasks.

| **First Word (W1)** | **Target – Phonological task** | | **Target – Semantic task** | |
| --- | --- | --- | --- | --- |
| arancia | costume | (mismatch) | cotone | (mismatch) |
| balcone | tronco | (mismatch) | pezza | (mismatch) |
| barba | gelato | (mismatch) | baffo | (match) |
| barca | marca | (match) | nave | (match) |
| bastone | leone | (match) | legno | (match) |
| bestiame | argento | (mismatch) | cappotto | (mismatch) |
| bicchiere | ventre | (mismatch) | camicia | (mismatch) |
| borsa | cotone | (mismatch) | costume | (match) |
| bottega | camicia | (mismatch) | ventre | (mismatch) |
| bottiglia | maniglia | (match) | vino | (match) |
| brodo | chiodo | (match) | minestra | (match) |
| cancello | carrello | (match) | porta | (match) |
| cappello | castello | (match) | giglio | (mismatch) |
| coda | biglietto | (mismatch) | latte | (mismatch) |
| collo | pollo | (match) | collana | (match) |
| coltello | ombrello | (match) | cucchiaio | (match) |
| cortile | latte | (mismatch) | biglietto | (mismatch) |
| farina | cascina | (match) | pane | (match) |
| fascina | gomma | (mismatch) | terrazzo | (mismatch) |
| foraggio | formaggio | (match) | pecora | (match) |
| foresta | cesta | (match) | albero | (match) |
| fucile | sedile | (match) | palco | (mismatch) |
| galleria | fumo | (mismatch) | quadro | (match) |
| gallo | lampada | (mismatch) | rotolo | (mismatch) |
| gettone | lupo | (mismatch) | fango | (mismatch) |
| giacca | baracca | (match) | cravatta | (match) |
| guanto | fango | (mismatch) | lupo | (mismatch) |
| limone | calza | (mismatch) | gelato | (match) |
| manica | orto | (mismatch) | aquila | (mismatch) |
| martello | gioiello | (match) | chiodo | (match) |
| moneta | seta | (match) | argento | (match) |
| montagna | castagna | (match) | disco | (mismatch) |
| motore | palestra | (mismatch) | parco | (mismatch) |
| mulino | camino | (match) | vento | (match) |
| nebbia | parco | (mismatch) | palestra | (mismatch) |
| neve | corda | (mismatch) | ghiaccio | (match) |
| onda | sponda | (match) | grano | (mismatch) |
| orecchio | specchio | (match) | naso | (match) |
| orologio | ghiaccio | (mismatch) | tetto | (mismatch) |
| osso | dosso | (match) | carne | (match) |
| palla | farfalla | (match) | armadio | (mismatch) |
| pallone | portone | (match) | lampada | (mismatch) |
| palo | schiena | (mismatch) | topo | (mismatch) |
| pancia | guancia | (match) | stomaco | (match) |
| patata | frittata | (match) | orto | (match) |
| penna | topo | (mismatch) | gomma | (match) |
| petto | schermo | (mismatch) | negozio | (mismatch) |
| piatto | orso | (mismatch) | unghia | (mismatch) |
| pino | lino | (match) | tronco | (match) |
| polso | negozio | (mismatch) | schermo | (mismatch) |
| ponte | fonte | (match) | torrente | (match) |
| quaderno | soffitta | (mismatch) | pugno | (mismatch) |
| rete | pugno | (mismatch) | soffitta | (mismatch) |
| riso | teatro | (mismatch) | pozzo | (mismatch) |
| rivista | pista | (match) | carta | (match) |
| roccia | goccia | (match) | frana | (match) |
| ruota | carota | (match) | carro | (match) |
| sabbia | poltrona | (mismatch) | denaro | (mismatch) |
| sacco | tabacco | (match) | tela | (match) |
| sale | viale | (match) | zucchero | (match) |
| sapone | bottone | (match) | vasca | (match) |
| sasso | grasso | (match) | sedile | (mismatch) |
| serpente | torrente | (match) | cartello | (mismatch) |
| spada | mosca | (mismatch) | lama | (match) |
| spiaggia | pozzo | (mismatch) | teatro | (mismatch) |
| sportello | cartello | (match) | cornice | (mismatch) |
| stella | sella | (match) | luna | (match) |
| suolo | verme | (mismatch) | uscio | (mismatch) |
| suono | trono | (match) | campana | (match) |
| tappeto | uscio | (mismatch) | verme | (mismatch) |
| tazza | terrazza | (match) | calice | (match) |
| tomba | stoffa | (mismatch) | marmo | (match) |
| toro | foro | (match) | nuvola | (mismatch) |
| torre | viso | (mismatch) | castello | (match) |
| uccello | sveglia | (mismatch) | carrozza | (mismatch) |
| uovo | statua | (mismatch) | gallina | (match) |
| vestaglia | tetto | (mismatch) | zampa | (mismatch) |
| vetro | carrozza | (mismatch) | sveglia | (mismatch) |
| volto | zucchero | (mismatch) | cucina | (mismatch) |
| vulcano | divano | (match) | fuoco | (match) |
